# Health and Its Relationship with Residential Relocations of Older People to Institutions versus to Independent Dwellings

**DOI:** 10.1007/s12062-017-9187-1

**Published:** 2017-06-04

**Authors:** Marieke van der Pers, Eva U. B. Kibele, Clara H. Mulder

**Affiliations:** 1 Faculty of Behavioural and Social Sciences, University of Groningen, Groningen, The Netherlands; 2 Statistisches Landesamt Bremen, Bremen, Germany; 3 Faculty of Spatial Sciences, Population Research Centre, University of Groningen, PO Box 800, 9700 AV Groningen, The Netherlands

**Keywords:** Residential relocations, Older people, Register data, Health measures, The Netherlands

## Abstract

Research into older people’s relocations to independent dwellings has largely remained separate from research into moves to institutions. Yet, both types of moves could be a response to health problems and to a certain extent they could be substitutes for each other. Using Litwak and Longino’s model of moves of older people, this study assesses the extent to which three commonly used health measures (limitations in activities of daily living [ADL], self-rated health, and the prevalence of [limiting] chronic conditions) predict older people’s moves to subsidized care institutions and elsewhere, in one multinomial logistic regression model. The data were derived from the POLS survey for the Netherlands (*N* = 8306) enriched with administrative data on subsequent moves. In line with Litwak and Longino’s model, the findings indicate that older people’s moves to institutions were more likely among those with more severe health problems, whereas moves elsewhere were more likely among those with moderate health problems. Among the three investigated health measures, limitations in ADL had the strongest predictive value, and was the only one for which the difference in effect between relocations to care institutions and relocations elsewhere was statistically significant.

## Introduction

At older age, changing residence, either to a care institution or to more suitable housing, can be a strategy to deal with the inability to remain living independently because of health problems. The research topic of residential relocations at older ages has thus far mainly been approached from two distinct angles, in different bodies of literature: Research on residential relocations and research on the use of residential care. Research on residential relocations has provided clear evidence that at older ages health is an important predictor of such relocations (Longino et al. 1991; Bradsher et al. 1992; De Jong et al. 1995; Friedman et al. 2016; for more references see Wilmoth 2010), although Longino et al. (2008) did not find an effect of self-rated health on older people’s non-local moves. Research on the use of residential care has shown that health is a very strong predictor of moves to residential care institutions (see reviews by Miller and Weissert 2000 and Luppa et al. 2010; Wilmoth 2010 refers to several studies from the 1990s).

Although the conditions for and consequences of living in a residential care facility differ from those associated with independent living, moves to either destination may be alternative responses to changing residential needs. For some older people, it may be possible to avoid or postpone a move to an institution if an adequate independent dwelling is available. To evaluate under what circumstances older people end up in an institution or rather in another independent dwelling it is necessary to set these alternatives next to each other in one analysis, and to investigate the extent to which health has an impact on older people’s changes of residence to care institutions versus elsewhere. Gaining insight into the different impact of health on the likelihood of these two types of moves, and into the likelihood of ending up in an institution versus a dwelling when moving, may help researchers and policymakers anticipate (future) residential needs in accordance with the current and expected health status of older people. Yet, hardly any previous research has included both types of residential relocations in one analysis of the likelihood of moving (exceptions are Miller et al. 1999, Bloem et al. 2008 and Van der Pers et al. 2015).^1^ The first aim of this paper is therefore to gain insight into the extent to which health predicts residential relocations of older people to care institutions versus elsewhere, by contrasting moves of either type not only to not moving but also to a move of the other type.

In the two bodies of literature, three health measures have commonly been used to predict older people’s moves: limitations in Activities of Daily Living (ADL), self-rated health, and the prevalence of chronic conditions. Empirical evidence indicates that older people who face limitations in ADL are more likely to move to institutions (Wolinsky et al. 1993; De Jong et al. 1995; Freedman 1996; Miller and Weissert 2000; Aguero-Torres et al. 2001; Thomése and Broese van Groenou 2006; Luppa et al. 2010). There is also some evidence of an increased likelihood of changing residence among those with ADL limitations (Granbom et al. 2014; Friedman et al. 2016; Rogerson et al. 1997 for moving closer to children; De Jong et al. 1995 found some evidence of an impact of ADL limitations on moves for health reasons). Similar results have been found for those who perceive their health as being poor (Freedman 1996; Miller and Weissert 2000; Luppa et al. 2010; McCann et al. 2011, for moves to institutions; Granbom et al. 2014, for changing residence). Experiencing (certain) chronic conditions or a long-term limiting illness has been found to be associated with a greater likelihood of moving to an institution (Aguero-Torres et al. 2001; Geerlings et al. 2005; Grundy and Jitlal 2007; McCann et al. 2011), although Puts et al. (2005) find no effect of chronic conditions after accounting for frailty.

Clearly, these three health measures are partly related to different dimensions of health. The use of these different measures therefore makes it difficult to compare results between studies and to assess whether differences in findings are caused by contextual differences or rather by using different health measures. It would be helpful to set analyses using the three measures next to each other. Indeed, some of the above-mentioned studies of moves to institutions use two of the three health measures, and Geerlings et al. (2005) use all three – but using multiple measures has rarely been done for moves elsewhere than to institutions. We were fortunate to have access to data in which all three health measures are available next to measures of both types of relocations. Our second aim is therefore to explore the extent to which the three health measures predict both types of residential relocations in different ways.

The data were derived from six versions of an annual nationally representative survey that includes health information (POLS) for the Netherlands. These surveys were enriched with administrative data to measure residential moves following each separate survey. The analyses are performed using multinomial logistic regression of the likelihood of moving to a care institution or elsewhere.

## Theoretical and Research Background

### Living Arrangements and Formal Care Use among Older People: the Context of the Netherlands

All citizens of the Netherlands have obligatory health insurance that covers both home care and institutional care (De Meijer et al. 2015). For a few decades now, care policy in the Netherlands has been aimed at stimulating people to live independently as long as possible (De Meijer et al. 2015). Admission to care institutions is needs-based and rules have become increasingly strict, leading to a decrease in the percentage of older people living in institutions since the 1990s (Verbeek-Oudijk et al. 2014; Swinkels et al. 2016).^2^ In contrast, home care use has grown (De Meijer et al. 2015). Suanet et al. (2012) found that the percentage of older people using formal home care was above-average in the Netherlands compared with other European countries, at around 16%. Given the strict needs assessment, one would expect some form of health problems to play a part in any move to an institution.

Among the vast majority of households of older people (age 55+) who lived in independent dwellings, around one third lived in a one-level dwelling in the early 2000s while over one fifth of households lived in dwellings that had some form of adaptations for older people (De Klerk 2004). For most rental dwellings adapted or suitable for older people needs assessment applies. Co-residence with children is rare; Suanet et al. (2012) describe the Netherlands as one of the two European countries in their study (next to Sweden) where attitudes towards co-residence are particularly unfavorable.

### Health as Predictor of Residential Relocations at Older Age

Health problems may lead people to experience practical difficulties in their immediate living environment which may require an adaptation of their housing or residential location. In their life course model of migration, Litwak and Longino (1987) distinguish between three types of moves among older people, the second and third of which are related to health. Their second type of move is a change of residence to more suitable housing when disabilities make everyday household tasks too difficult to perform and when an adjustment of the current dwelling is not possible or does not satisfy needs. The third type is a move to a residential care facility, which becomes likely when adequate resources to receive support and care at home are lacking or are not sufficient. Following their reasoning, health should play a part in relocations to institutions as well as elsewhere. Overall, moves of older people are indeed frequently health-related (Longino et al. 1991; Bradsher et al. 1992; De Jong et al. 1995; Wilmoth 2010); and poor and declining health is also an important predictor of institutionalization (Wolinsky et al. 1993; Miller and Weissert 2000; Puts et al. 2005; Geerlings et al. 2005; Thomése and Broese van Groenou 2006; Bloem et al. 2008; Luppa et al. 2010; Grundy 2011).

In line with Litwak and Longino’s (1987) model, one would expect severe health problems mainly to lead to moves to institutions, whereas moderate health problems would mainly lead to moves elsewhere. The evidence from the few existing studies that set the two types of moves next to each other indeed points in that direction. Bloem et al. (2008) conducted a multinomial regression on relocations of people aged 54 and older from regular housing to regular, adapted and institutional residences. Using one composite measure of health based on five different more detailed measures, they found that a decline in health had a significant effect on moves to care institutions and they also showed that this effect was stronger when initial health was poor (Bloem et al. 2008). They however found no significant impact of declining health on moves to regular and adapted housing. Van der Pers et al. (2015) used ‘closeness to death’ as proxy of severe health problems. They found that people who were close to death were more likely to change residence, with a stronger effect on moving to a care institution than on moving elsewhere.

There are two reasons for expecting a greater impact of health for women than for men. First, women tend to suffer more from (long term) multiple chronic disabilities, while men tend to suffer more from fatal conditions (Leveille et al. 2000), and this difference might not adequately be captured by the health measures we use. Furthermore, married men receive more home care from their partners than married women (e.g. Glauber 2017), which could facilitate a prolonged stay at home. At the same time, women are generally better able to take care of themselves and can rely on larger and more supportive social networks than men (Van Gaalen and Dykstra 2006; McLaughlin et al. 2010), which would lead to a smaller impact of health for women than men.

### Different Health Measures Predicting Older People’s Residential Relocations

Various health measures have been used as predictors of residential relocations at older ages. This partly reflects the fact that health is a multidimensional and dynamic concept, which leads to variations in definitions of health status. Furthermore, many studies on older people’s moves use data that have been collected for a broader purpose, and such data often contain only one health measure or just a few to choose from.

Following Litwak and Longino’s (1987) model, for research into residential relocations the health problems that need to be measured are those that are associated with disabilities in performing regular household tasks and moving around in and around the house. The commonly used measures ‘limitations in activities of daily living’, ‘self-rated health’, and ‘the prevalence of (limiting) chronic conditions’ are incorporated in this study. Naturally, the three measures overlap, but there are also differences.

Other commonly used predictors, mainly of institutionalization, are cognitive impairment (Aguero-Torres et al. 2001; Miller and Weissert 2000; Gaugler et al. 2009; Luppa et al. 2010) and limitations in instrumental activities of daily living (IADL) (Wilmoth 2010; Luppa et al. 2010; Miller and Weissert 2000; Freedman 1996; Hallberg and Lagergren 2009). Data restrictions do not allow us to incorporate these.

### Difficulties in Activities of Daily Living (ADL)

The inability to carry out functional tasks at the personal level is usually operationalized by a standardized measure of biological and psychosocial functioning, also called ‘activities of daily living’ (ADL). Difficulties in performing ADL are associated with the use of long term care services (Borrayo et al. 2002). They also predict older people’s residential relocations (De Jong et al. 1995; Rogerson et al. 1997; Wilmoth 2010). Limitations in ADL indeed also predict relocations to such institutions (Freedman 1996; Miller and Weissert 2000; Thomése and Broese van Groenou 2006; Luppa et al. 2010; Wilmoth 2010; Gaugler et al. 2009; Hallberg and Lagergren 2009). From a theoretical point of view, difficulties in ADL seem to come closest to the kind of disabilities Litwak and Longino (1987) referred to in their model of older people’s moves. For example, when difficulties in climbing the stairs make it more difficult to move around in the house, some people may move to a one-level house or apartment. With more severe practical limitation(s), such a move will not be sufficient and a move to a residential care institution may be required.

### Self-Rated Health

Self-rated health (SRH, also denoted as perceived health) measures how a person perceives his or her current physical and mental well-being and is related to overall functioning (Lee 2000) and health care utilization (Idler and Benyamini 1997). Poor and declining SRH predicts moves of older people (Wilmoth 2010). Granbom et al. (2014) found that SRH predicts moves to special needs housing, and others have shown that it is an important predictor of institutionalization (Freedman 1996; Luppa et al. 2010; McCann et al. 2011). It should be noted that older people may report better health than younger people in similar conditions may do, because they rate their health relative to the health of their peers, or consider health deterioration as a normal consequence of aging and not as a symptom of disease (Leinonen et al. 1997; Bardage et al. 2005). Furthermore, poor self-rated health does not necessarily come with disabilities.

### Chronic Conditions

The presence of chronic conditions is frequently used to predict older people’s moves to institutions (Aguero-Torres et al. 2001; Geerlings et al. 2005; Puts et al. 2005; Miller and Weissert 2000). Chronic conditions are related to functional limitations (Verbrugge and Jette 1994) which may lead to changes in residential needs. However, some chronic conditions will lead to disabilities, but other conditions may not. For example, someone with diabetes may not experience any practical difficulties whereas someone with severe Parkinson’s disease may only find adequate care and support in a residential care facility. Following Grundy and Jitlal (2007) and McCann et al. (2011), we therefore use an extended measure of chronic conditions that distinguishes conditions that are accompanied by physical limitations in daily activities at home from those that are not.

## Data and Methods

### The Dataset and Study Population

Data were extracted from six versions of the cross-sectional annual Dutch health survey ‘POLS’ (in Dutch: *Periodiek Onderzoek Leefsituatie*), conducted by Statistics Netherlands (CBS 2013). Each survey comprises a random sample of the non-institutionalized population. From the surveys conducted in the years 2003–2008, we extracted those 8427 people who were aged 65 and older and whose address one year after the survey could be traced in the population or institutionalization register; this implies that those who died or emigrated in the meantime were not included. The number of respondents per survey ranged between 1286 in 2007 and 1508 in 2005. Of the 8427, 121 people were excluded owing to missing data on the selected health indicators, education or homeownership,^3^ leading to a final sample size of 8306, with mean age 73.8 and maximum age 99. With a unique data key provided by Statistics Netherlands respondents of each survey were matched to two administrative datasets: the population register which contains residential addresses (CBS 2010a) and a dataset containing information about admissions to subsidized residential care facilities (*Centraal Administratie Kantoor-Zorg met Verblijf*) (CBS 2012).

#### Dependent Variable

The response categories of the dependent variable are: no residential relocation (0, reference category), residential relocation to a care institution (1), and residential relocation elsewhere (2). A move elsewhere was measured as a change of address between the time of the POLS survey (t0) and exactly one year later (t + 1). Thus, if the respondent made multiple moves within a year only the last was recorded. Because no information was available about changes in health status after the survey and health status can change rapidly over time, we did not think it was appropriate to use information about changes of address more than one year from the survey. Relocations to care institutions were derived from data on the start and duration of stays in subsidized residential care facilities. Following Van der Pers et al. (2015) we excluded all types of residential care facilities that primarily provide rehabilitative services, such as hospitals and revalidation centers. We considered a person to have relocated to a care institution when the duration of stay was at least 90 days between t0 and t + 1, regardless of whether a change of address was registered. It should be noted that the database does not cover unsubsidized residential care facilities. However, in the context of the Netherlands, this does not lead to a problematic bias because the vast majority of the residential care facilities are subsidized (Mestheneos and Triantafillou 2005; RIVM 2013).

#### Key Explanatory Variables: Three Health Measures

The POLS survey contains information about ten activities of daily living: eating and drinking; getting into and out of a chair; getting into and out of bed; dressing; moving to another room at the same floor; moving up and down the stairs; entering and leaving the house; moving around outside the house; washing the face and hands; washing the body completely. For each activity the respondent reports whether it can be performed without any effort (score 0), with some effort (1), with major effort (2), or only with help from others (3). We created the variable *difficulties in ADL* by grouping the added scores on the ten items into four categories; score 0, score 1–3, score 4–10 and score 11 or more (maximum score 30). *Self-rated health (SRH)* was measured using the single-item question ‘how is your health in general?’. The answers were grouped into very good/good, moderate and poor/very poor self-rated health. The variable *chronic conditions* is a composite measure based on a question whether the respondent suffered from one or more chronic illnesses, conditions or handicaps (yes or no^4^) and a question about the extent to which the respondent experiences difficulties in performing daily activities at home because of the chronic condition(s). The categories for this variable are ‘no chronic conditions at all’, ‘chronic condition(s) but no limitations’, ‘chronic condition(s) and moderate limitations’, ‘chronic condition(s) and severe limitations’. It should borne in mind that owing to the construction of this measure, limitations in daily activities that are not related to a chronic condition are not represented in it. The grouping of all three variables was based on how well a particular grouping discriminated between the categories of the dependent variable.

#### Control Variables

Most control variables were derived from the POLS survey, and all were measured at baseline. Among the older population, the propensity to change residence increases with age, in particular to care institutions (Van der Pers et al. 2015). We categorized *age* as ‘65–74 years’, ‘75–79 years’ and ‘80 years and older’. Because older people without a partner are more likely to move (Longino et al. 1991; Chevan 1995; De Jong et al. 1995; Wilmoth 2010) and to become institutionalized (Miller and Weissert 2000; Luppa et al. 2010), the variable *partnership status* reflects whether a person lived with a partner or not. The geographic proximity of children also predicts whether older people move (De Jong et al. 1995; Smits 2010). *Proximity of children* was constructed using the population register and record linkage of parents and children (CBS 2010b), and was categorized as having a child living within 20 km Euclidean distance, not having a child within 20 km, and not having children at all (see also Van der Pers et al. 2015).

In the general population, higher levels of education lead to more mobility (Börsch-Supan 1990). Older people with a higher educational level generally also have a higher income and more assets, which supports the realization of a desire to change residence on the one hand, but to buy private assistance at home on the other hand. Review studies point to non-significant and inconclusive findings of educations on moves to care institutions (Miller and Weissert 2000; Luppa et al. 2010). The variable *education* was classified into three educational levels: low (primary school and lower vocational education), middle (secondary school and intermediate vocational education) and high (higher vocational education and university). As being a homeowner reduces older people’s likelihood of changing residence (Abramsson and Andersson 2012) and institutionalization (Miller and Weissert 2000; McCann et al. 2012), the variable *homeownership* differentiates between owner-occupiers and renters. Finally, adaptive strategies may vary by the availability of (in)formal care in nearby surroundings which may lead to differences in the likelihood of changing residence according to level of urbanization. *Degree of urbanization* was derived from the population register and based on address density at the neighborhood level (urban: 1500 or more addresses per km^2^, less urban: 500 to 1500 addresses, rural: fewer than 500 addresses per km^2^).

### Analytical Strategy

We estimated multinomial logistic regression models of having moved to a care institution or elsewhere, one year after baseline. We ran one model without any health measure (Model 0), and separate models for each of the three health measures and stratified them by gender (Models A, B and C). We tested whether each separate model A, B and C was significantly different from Model 0 by comparing the goodness of fit through a likelihood ratio test.

The presented models have ‘no residential relocation’ as reference category. To evaluate the effects on moving to a care institution compared with moving elsewhere we also present the significance levels of models with ‘moving elsewhere’ as a reference category. We furthermore performed statistical tests to evaluate whether the effects differ between men and women.

Interactions between the health measures and partnership status were explored, but owing to the small number of cases, the outcomes were considered as unreliable.

## Results

### Descriptives

One year after baseline 3,6% of the men and 4,9% of the women had changed residence, of whom 22 and 42% moved to a care institution (Table 1). It should be noted that the number of moves to care institutions was small, particularly among men (*n* = 29). Moves to care institutions were particularly frequent among those with severe difficulties in ADL, whereas moves elsewhere were more frequent among those with fewer difficulties in ADL. Differences in the likelihood of moving among the categories of the other health measures are also found, but these are less pronounced. Women moved more frequently than men; this gender difference was greater for moves to institutions than for moves elsewhere.

**Table 1:** Frequency distribution of independent variables, by type of residential relocation, in percentages

	Men			Women		
	% in sample	Relocation to institution	Relocation elsewhere	% in sample	Relocation to institution	Relocation elsewhere
Age						
65–69 years	62.4	0.3	2.7	55.2	0.5	3.0
70–79 years	20.9	0.5	2.6	21.8	1.9	2.9
80 years and older	16.7	2.7	3.5	23.0	5.8	2.6
Partnership status						
Living with partner	79.4	0.4	3.0	52.2	1.0	2.2
Living without partner	20.6	2.1	2.2	47.8	3.2	3.6
Proximity of children						
Having a child living within 20 km	74.7	0.8	2.8	76.1	1.8	2.8
Not having a child living within 20 km	13.4	0.4	4.4	12.8	1.4	2.9
No children at all	11.9	1.1	1.3	13.2	3.5	3.2
Educational level						
Low	44.8	0.9	2.6	64.1	2.2	2.7
Middle	34.8	0.9	3.5	26.7	1.9	3.3
High	20.4	0.4	2.1	9.2	1.2	3.1
Home ownership						
No	45.7	1.2	2.9	55.7	2.9	3.2
Yes	54.3	0.4	2.7	44.3	1.0	2.4
Degree of urbanisation						
Urban	38.7	0.8	2.4	41.9	2.5	3.2
Less urban	22.2	0.6	3.0	21.2	1.3	2.6
Rural	39.0	0.9	3.1	36.9	1.9	2.7
Difficulties in ADL						
Score 0	67.8	0.2	2.6	51.8	0.5	2.4
Score 1–3	17.9	0.9	2.4	23.1	1.9	3.3
Score 4–10	10.3	2.6	4.7	18.3	3.8	3.8
Score 11 or more	4.0	4.7	3.3	6.8	9.6	2.9
Self-rated health						
Very good/good health	62.7	0.4	2.5	55.2	1.1	2.4
Moderate health	29.3	1.5	3.5	31.7	3.2	3.6
Poor/very poor health	8.1	1.3	2.7	8.8	4.7	4.7
Chronic conditions						
No chronic condition(s)	47.8	0.5	2.1	40.8	1.1	2.7
Chronic condition(s), no limitations	20.3	0.4	3.0	15.3	0.6	2.0
Chronic condition(s), moderate limitations	14.4	2.4	3.9	20.9	4.3	3.8
Chronic condition(s), severe limitations	16.7	0.6	3.7	22.7	1.7	3.0
N	3.726	29	108	4.580	93	123
Percentage movers		0.8	2.8		2.0	2.9
Percentage to institution/elsewhere among movers		21.6	78.4		41.3	58.7

Statistics Netherlands (2013, 2010a, b, CBS 2012)

### Different Health Measures Predicting Older People’s Moves: Multinomial Regression Results

Compared with Model 0 that does not include a health measure, the inclusion of any of the three health measures significantly improves the prediction of older people’s residential relocations (Table 2). Yet, for men the inclusion of SRH leads to an only marginally significant improvement (p < 0.10). The results also show that of the three health measures, the measure difficulties in ADL has the greatest predictive power. For women the predictive power of the models including SRH and chronic conditions is similar, whereas for men the model with chronic conditions has a lower predictive power than the model with SRH.

**Table 2:** Multinomial logistic regression of residential relocations of people aged 65 years and older at baseline, stratified by gender. Effects from separate models including one health measure each

	Men					Women				
	Relocation to institution versus no relocation		Relocation elsewhere versus no relocation		Institution versus elsewhere^a^	Relocation to institution versus no relocation		Relocation elsewhere versus no relocation		Institution versus elsewhere^a^
	*B*	*S.E.*	*B*	*S.E.*	*p*	*B*	*S.E.*	*B*	*S.E.*	*p*
Model 0: Without health indicator										
*LL (df)*	−616.37 (20)					−986.19 (20)				
*Pseudo R* ^*2*^	0.048					0.061				
Model A: Difficulties in ADL										
Score 0	0		0			0		0		
Score 1–3	0.872	0.593	−0.101	0.238	0.138	0.998	0.384 ***	0.390	0.225	0.169
Score 4–10	1.873	0.541 ***	0.644	0.283 **	0.042	1.524	0.365 ***	0.566	0.238 **	0.026
Score 11 or more	2.334	0.603 ***	0.282	0.483	0.007	2.367	0.376 ***	0.359	0.378	0.000
*LL(df)*	−603.86 (26)					−958.69 (26)				
*Pseudo R* ^*2*^	0.067					0.087				
*Model A* versus *Model 0 (LR; Δdf; sign)*	(25,01; 6; ***)					(55,00; 6; ***)				
Model B: Self-rated health										
Very good/Good health	0		0			0		0		
Moderate health	1.060	0.427 **	0.323	0.215	0.121	0.740	0.249 ***	0.312	0.196	0.171
Poor/Very poor health	1.098	0.623 *	0.097	0.387	0.169	1.252	0.314 ***	0.675	0.275 **	0.159
*LL (df)*	−611.67 (24)					−974.58 (24)				
*Pseudo R* ^*2*^	0.055					0.072				
*Model B* versus *Model 0 (LR; Δdf; sign)*	(9,39; 4; *)					(23,23; 4; ***)				
Model C: Chronic conditions										
No chronic condition(s)	0		0			0		0		
Chronic condition(s), no limitations ^b^	−0.160	0.675	0.325	0.269	0.502	−1.090	0.538 **	−0.356	0.306	0.234
Chronic condition(s), moderate limitation	1.475	0.450 ***	0.631	0.280 **	0.108	0.628	0.250 **	0.334	0.227	0.377
Chronic condition(s), severe limitations	0.173	0.610	0.536	0.270 **	0.584	−0.067	0.306	0.076	0.232	0.707
*LL(df)*	−606.33 (26)					−974.76 (26)				
*Pseudo R* ^*2*^	0.063					0.072				
*Model C* versus *Model 0 (LR; Δdf; sign)*	(20,08; 6; **)					(22,86; 6; ***)				

SE: standard error. Reference group is no residential relocation. Controlled for the variables age, partnership status, proximity of children, education, homeownership and degree of urbanisation. Statistics Netherlands (2013, 2010a, b, CBS 2012)

*** *p* < 0.01, ** *p* < 0.05, * *p* < 0.1

^a^Significance levels for models with reference category ‘residential relocation-elsewhere than to care institution’

^b^the effects of ‘chronic condition(s), no limitations’ for men and women moving elsewhere are marginally significantly different from each other (*p* = 0,0949)

Men and women with greater difficulties in ADL, poorer SRH, and limiting chronic conditions are more likely to change residence to both destinations than men and women without or with less severe health problems. The higher the score on difficulties in ADL, the greater the likelihood of a move to an institution is estimated to be. For moves elsewhere there is no such monotonic pattern: we find significant positive effects of having rather severe limitations in ADL (score 4–10), but smaller and insignificant effects of less (score 1–3) and more severe (score 11 or more) limitations. We also find a significant positive effect of poor and very poor SRH for women on moving elsewhere, whereas for men having a chronic condition with moderate or severe limitations has a significant positive effect on moving elsewhere. The effects of ‘chronic condition(s), no limitations’ on moving elsewhere are mixed and partly counter-intuitive. This can possibly be explained from the heterogeneity of the reference category: part of those without chronic conditions have limitations not related to a chronic condition.

The effects of having more severe difficulties in ADL (scores 4–10 and 11 or more) on moving to a care institution are significantly stronger than its effects on moving elsewhere. For SRH and chronic conditions the effects are also estimated to be stronger for moves to a care institution than for moves elsewhere, but the difference is not statistically significant. This implies ‘difficulties in ADL’ is the only health indicator for which our data allow us to draw conclusions on older people’s choices between moving to an institution and moving to another independent dwelling.

### Control Variables

With increasing age women are more likely to move to a care institution and less likely to move elsewhere (Table 3). Men aged 80 years and older are more at risk of becoming institutionalized than men aged 65–69, but we do not find a significant age effect for men on moving elsewhere.

**Table 3:** Multinomial logistic regression of residential relocations of people aged 65 years and older at baseline, stratified by gender. Effects of control variables (model A)

	Men					Women				
	Relocation to institution versus no relocation		Relocation elsewhere versus no relocation		Institution versus elsewhere^a^	Relocation to institution versus no relocation		Relocation elsewhere versus no relocation		Institution versus elsewhere^a^
	*B*	*S.E.*	*B*	*S.E.*	*p*	*B*	*S.E.*	*B*	*S.E.*	*p*
Constant	−6.576	0.731 ***	−3.372	0.290 ***	0.000	−6.577	0.499 ***	−4.011	0.285 ***	0.000
Age										
65–69 years	0		0			0		0		
70–79 years	−0.016	0.624	−0.092	0.265	0.909	0.783	0.372 **	−0.236	0.230	0.018
80 years and older	1.265	0.458 ***	0.256	0.266	0.055	1.638	0.330 ***	−0.490	0.247 **	0.000
Partnership status										
Living with partner	0		0			0		0		
Living without partner ^b^	1.046	0.399 ***	−0.288	0.279	0.006	0.440	0.263 *	0.528	0.193 ***	0.785
Proximity of children										
Having a child living within 20 km	0		0			0		0		
Not having a child living within 20 km	−0.269	0.760	0.591	0.256 **	0.281	−0.018	0.390	0.282	0.274	0.523
No children at all	0.047	0.519	−0.675	0.432	0.281	0.458	0.275 *	0.016	0.265	0.238
Educational level										
Low	0		0			0		0		
Middle	0.403	0.410	0.280	0.222	0.789	0.238	0.266	0.289	0.207	0.949
High	−0.501	0.788	−0.292	0.319	0.805	−0.178	0.494	0.186	0.323	0.533
Home ownership										
No	0		0			0		0		
Yes	−0.592	0.448	−0.110	0.215	0.329	−0.486	0.277 *	−0.243	0.200	0.474
Degree of urbanisation										
Urban	0.110	0.555	−0.193	0.269	0.621	0.589	0.324 *	0.170	0.243	0.295
Less urban	0		0			0		0		
Rural	0.421	0.547	0.032	0.256	0.516	0.615	0.344 *	0.137	0.254 ***	0.259
Model summary										
N	3726					4580				
LL (df)	−603.86 (26)					−958.69 (26)				
Pseudo R^2^	0.066					0.086				

SE: standard error. Reference group is no residential relocation. Variable difficulties in ADL controlled for. Statistics Netherlands (2013, 2010a, b, CBS 2012).

*** *p* < 0.01, ** *p* < 0.05, * *p* < 0.1.

^a^ Significance levels for models with reference category ‘residential relocation-elsewhere than to care institution’

^b^ the effects of ‘living without a partner’ for men and women moving elsewhere are significantly different from each other (*p* = 0,0163)

For men living without a partner increases the likelihood of moving to an institution but not of moving elsewhere, whereas women without a partner are significantly more likely to move elsewhere. Men without a child living close are more likely to move elsewhere than men who have a child living close. Childless women are more likely to become institutionalized.

We do not find any other statistically significant effects for men, but for women we do. The findings indicate that highly educated older women are less likely to become institutionalized than those who are less educated. We find a significant negative effect of homeownership on the institutionalization of women. Furthermore, women living in more urban and more rural areas are more likely to move to a care institution than older women living in suburban areas.

## Discussion

Older people use adaptive strategies in order to deal with health problems that make it more difficult to remain living independently and are therefore more likely to change residence when they face health problems. We acknowledge that the conditions for and consequences of living in a residential care facility differ from those associated with independent living, and in this study we therefore examined the different impact of health on older people’s residential relocations to care institutions and elsewhere. The findings confirm that health is an important predictor of older people’s moves and that more severe health problems make older people more likely to change residence, with stronger effects on relocations to a care institution than on relocations elsewhere.

Of the three commonly used health measures ‘limitations in Activities of Daily Living’, self-rated health and chronic conditions, limitations in ADL has the most pronounced effects for both older men and older women. Moreover, it is the only measure that was shown to have a significantly different impact on moves to institutions than on moves elsewhere. Although the measure self-rated health does contribute to predicting relocations of older people, the effects are not particularly strong and are not found to differ by destination. In a similar manner, neither does the measure chronic conditions distinguish between the two destinations, and its effects are small. It should be borne in mind that a large share of older people experience some kind of chronic condition, and many of these conditions are not accompanied by functional limitations in daily tasks at home.

The greater predictive power of the measure limitations in ADL is in line with Litwak and Longino’s (1987) model of moves of older people. Conceptually, it would be difficulties accessing, moving around in, cleaning and maintaining a home that would lead people to move to another home or to an institution. The indicator limitations in ADL has been designed precisely to measure such difficulties. We recommend using this measure in future research on residential relocations at older ages when it is available.

A novelty of this study is the treatment of moving to institutions and moving elsewhere as multiple risks in one analysis. This analytical strategy helped reveal how health, and also age, are related to these two moving destinations in different ways. Another strength was the record linkage of administrative data to survey data. This linkage enabled us to incorporate residential relocations to care institutions that are often not recorded in surveys. It also enabled us to add longitudinal characteristics on changes of residence to cross-sectional survey data on health. With this linkage we could approach the impact of health on residential moves prospectively. This approach is cost-effective and reduces selectivity bias due to attrition problems that occur when similar data would be collected in panel surveys. With this approach we have also been able to overcome the problem of limited information on health and other independent variables that Van der Pers et al. (2015) faced when making use of register data only.

Besides these strengths, our approach also has its weaknesses. First of all, the analyses were based on a small number of moves which likely has led to the insignificant effects of some control variables. Particularly the number of older men moving to a care institution was small (*n* = 29). Yet, the findings on the impact of health can be judged as reliable enough. Secondly, by using survey data as a basis for constructing the study population, the effects of health on residential relocations are most likely underestimated because those with very poor health status may not to have participated in the survey. Underestimation could also have occurred because we considered residential relocations in only a short time span. The effects of health could also be underestimated in the situation when older people without health problems moved because of the health problems of their partner. Unfortunately information about the health of partners was not available in the survey data. The inclusion of such information could have led to a greater predictive power of the models.

Another important explanatory factor that could not be included is changing health status. Bloem et al. (2008) found that changes in health had an additional effect on older people’s moves over and above health itself. We were not able to include information about changes in health, but it could be interesting to investigate whether particular types of health shocks lead to a greater likelihood of moving to a care institution or elsewhere. Furthermore, others have found predictive effects of cognitive impairment and limitations in IADL on institutionalization (Aguero-Torres et al. 2001; Freedman 1996; Hallberg and Lagergren 2009; Luppa et al. 2010; Miller and Weissert 2000; Speare et al. 1991) and relocations elsewhere (Longino et al. 1991; De Jong et al. 1995; Wilmoth 2010). Using these more detailed measures could generate insight into the extent to which the effects of these measures would differ from the effect of limitations in ADL.

It would also help to include information about the suitability of the current dwelling for those with limitations. For example, it would be helpful to know whether the current dwelling is accessible without climbing stairs, and whether there are stairs inside the dwelling.

Support provision is arranged in different ways in different countries. The findings may therefore be specific to the context of the Netherlands, where formal care at home and residential care institutions are subsidized by the state, and access to such institutions is limited to people with physical limitations. It could be helpful to replicate this research in contexts that differ from the Netherlands in these respects. In any case, we hope to have contributed to the literature on older people’s residential relocations by demonstrating how the determinants of relocations differ between moves to care institutions and moves elsewhere and by showing how three common health measurements differ in their capability of predicting these two types of relocations.

## Footnotes

1 Furthermore, De Jong et al. (1995) distinguished ‘move to a nursing home’ from several other motives for moving.

2 Swinkels et al. (2016) found a decrease from 4.7% in 1992 to 1.5% in 2009 in data from the Longitudinal Ageing Study Amsterdam.

3 There were no missing values on the other variables.

4 No further questions were asked about which conditions the respondent suffered from.
